# The prevalence and clinical significance of Presymptomatic COVID-19 patients: how we can be one step ahead in mitigating a deadly pandemic

**DOI:** 10.1186/s12879-021-05849-7

**Published:** 2021-03-09

**Authors:** Juen Kiem Tan, Dalleen Leong, Hemalatha Munusamy, Nor Hazwani Zenol Ariffin, Najma Kori, Rozita Hod, Petrick Periyasamy

**Affiliations:** 1 Infectious Disease Unit, Department of Internal Medicine, Faculty of Medicine, Universiti Kebangsaan Malaysia, Jalan Yaacob Latif, Bandar Tun Razak, 56000 Kuala Lumpur, Malaysia; 2 Department of Community Health, Faculty of Medicine, Universiti Kebangsaan Malaysia, Jalan Yaacob Latif, Bandar Tun Razak, 56000 Kuala Lumpur, Malaysia

**Keywords:** Coronavirus disease 2019, COVID-19, SARS-CoV-2, Presymptomatic

## Abstract

**Background:**

Presymptomatic COVID-19 patients have been identified as a major stumbling block in efforts to break the chain of transmission. Studies on temporal dynamics of its shedding suggests it peaks 1–2 days prior to any symptom onset. Therefore, a large proportion of patients are actively spreading the disease unknowingly whilst undetected. However, lengthy lockdowns and isolation leads to a host of socioeconomic issues and are impractical. Conversely, there exists no study describing this group and their clinical significance despite their key role in disease transmission.

**Methods:**

As a result, we devised a retrospective study to look at the prevalence of presymptomatic patients with COVID-19 from data sourced via our medical records office. Subsequently, we identify early indicators of infection through demographic information, biochemical and radiological abnormalities which would allow early diagnosis and isolation. In addition, we will look into the clinical significance of this group and their outcome; if it differs from asymptomatic or symptomatic patients. Descriptive statistics were used in addition to tabulating the variables and corresponding values for reference. Variables are compared between the presymptomatic group and others via Chi-square testing and Fisher’s exact test, accepting a *p* value of < 0.05 as significant.

**Results:**

Our analysis shows a higher proportion of presymptomatic patients with atypical symptoms like chest pain while symptomatic patients commonly present with respiratory symptoms like cough and shortness of breath. Besides that, there were more females presenting as presymptomatic patients compared to males (*p* = 0.019) and these group of patients were likely to receive treatment (*p* < 0.001). Otherwise, we were not able to identify other statistically significant markers suggesting a patient is presymptomatic.

**Conclusion:**

As we have little means of identifying these silent spreaders, it highlights further the importance of general measures implemented to stop COVID-19 transmission like social distancing, face mask, and widespread testing.

## Background

The start of the new decade would undoubtedly be remembered as a time when a global pandemic had brought most parts of the world to a complete standstill. The coronavirus disease 2019 (COVID-19) first reported a year ago in Wuhan, China has since infected sixty-two million globally, claiming over 1.4 million lives at the time of writing [1]. The effectiveness of symptom-based isolation has been curtailed by the presence of presymptomatic and asymptomatic patients, leading to multiple waves of infections and difficulty to curb its spread [2, 3].

Presymptomatic is defined as the presence of illness before the appearance of symptom. Published data have shown that COVID-19 is transmissible via presymptomatic patients, as well as growing data suggesting asymptomatic transmission, forcing healthcare providers to formulate various strategies in disease control [2–4]. He and Lau et al. had demonstrated that the highest viral load in throat swabs are at symptom onset and may have peaked 1–2 days earlier in presymptomatic stage, while pooled data of 1251 cases suggests that presymptomatic infectious rates could be as high as 68% of total infections [5, 6]. Therefore, failing to address this population would explain our failure in containment and mitigation of the pandemic.

We acknowledge that movement restrictions are not a feasible long-term plan with detrimental socioeconomic effects. However, failing to enforce social distancing amongst the public would lead to irrepressible infection rates. Therefore, in this study, we hope to re-examine our local demographics, presentations and baseline investigations, observing for possible patterns or early abnormalities that may suggest patients infected with COVID-19 may be at presymptomatic stage, hoping to get one step ahead of the pandemic and minimizing its socioeconomic impact.

## Methods

We carried out a retrospective single centre study between 17th March 2020 and 26th April 2020 at the National University of Malaysia Medical Centre. Our centre is based in Cheras, one of four red zone districts in Kuala Lumpur, declared since 6th April 2020 when it recorded more than 41 new confirmed case per day. Participants recruited were confirmed COVID-19 patients referred for tertiary care from primary and private healthcare centres as well as quarantine facilities having tested positive via qualitative reverse transcription polymerase chain reaction (RT-PCR) from nasopharyngeal and/or oropharyngeal swab, with cycle threshold value set at 45, targeting the RNA-dependent RNA polymerase (RdRp) and envelope (E) genes.

Data were sourced via our centre’s medical records department manually as well as additional history from patients still admitted and recovering as required. Demographic details collected include age, gender, background medical illnesses, cigarette smoking, alcohol consumption, presence of symptoms prior to and/or during admission, blood abnormalities, chest x-ray and/or chest computed tomography changes, treatment given and classifying patients to three groups; presymptomatic, symptomatic or true asymptomatic; with further note on atypical or paucisymptomatic presentation.

In Malaysia, patients with COVID-19 are divided to five clinical categories to depict the severity of the case; 1-asymptomatic, 2-symptomatic, 3-evidence of pneumonia, 4-oxygen supplement requirement, and 5- intubated and/or multiorgan failure, Afebrile and febrile patients are labelled as ‘A’ and ‘B’ respectively within the clinical category (eg. 2A = afebrile patient with symptoms, 2B = febrile patient with symptoms). These details including the highest documented modified early warning score (MEWS); which is used to guide frequency of vital sign monitoring; were documented.

Patients were classified as symptomatic if they have symptoms prior to a positive test result and true asymptomatic for those who had developed no symptoms throughout the infection [7]. Patients are labelled presymptomatic if they were asymptomatic prior to testing but developed symptoms within 14 days after testing positive based on studies showing its incubation period extending to 15 days. However, we also included those who presented with symptoms beyond that as similar studies have suggested that 101 out of every 10,000 cases may develop symptoms beyond 14 days [8, 9]. They were further classified to typical symptoms; fever, cough, shortness of breath or exertional dyspnea including bendopnea (previously coined for heart failure but significant among our demographics; representing a Muslim majority), atypical symptoms (chest pain, headache, gastrointestinal complaints, etc.) or paucisymptomatic having 1–2 minor symptoms [10–12].

Statistical analysis was performed with SPSS Version 26.0 statistic software package. To summarise the data, we used descriptive statistics in addition to tabulating the variables and corresponding values for reference. Variables are compared between the presymptomatic group and others (including symptomatic and true asymptomatic group) via Chi-square testing, Fisher’s exact test and one-way ANOVA accepting a *p* value of < 0.05 as significant.

## Results

A total of 205 patients with COVID-19 have been treated at our centre. We included 199 patients in our study, having excluded six patients as key data were lacking in their medical records. The mean age of patients was 34 ± 16 (SD) years ranging from 2 to 91 years old. A male preponderance was observed making up 73.9% of patients. Mean body mass index (BMI) was 25.3 ± 6.3 (SD) ranging from 15 to 52. Besides that, 53 patients (26.6%) in our cohort have comorbidities; mainly hypertension, diabetes mellitus and dyslipidemia. Interestingly, there were no patients with background chronic obstructive pulmonary disease.

From our data, most patients (95%) were diagnosed positive through screening (via contact tracing, high risk groups). In addition, 31 patients (15.6%) had history of recent overseas travel. Eventually, 196 patients were discharged well, one patient with multiple comorbidities succumbed to the disease and two patients were transferred out to a nearby hospital catering for infectious diseases prior to our centre’s active management of COVID-19 patients. Our sample’s demographic details are summarised in Table 1.

**Table 1:** Demographic information of our cohort of COVID-19 patients (*n* = 199)

Demographics	n (%)
Age (years old)	
< 10	1 (0.5)
10–19	27 (13.6)
20–29	78 (39.2)
30–39	34 (17.1)
40–49	24 (12.1)
50–59	15 (7.5)
60–69	12 (6)
70–79	6 (3)
80–89	1 (0.5)
> 90	1 (0.5)
Gender	
Male	147 (73.9)
Female	52 (26.1)
BMI *	
< 18.5	7 (6.4)
18.5–22.9	40 (36.7)
23–26.9	32 (29.4)
> 27	30 (27.5)
Cigarette smoking *	
Active	15 (12.1)
Non-smoker	101 (81.5)
Reformed	8 (6.4)
Alcohol consumption *	7 (11.3)
Comorbidites	53 (26.6)
Diabetes	13
Hypertension	26
Dyslipidaemia	10
Ischaemic heart disease	4
Chronic kidney disease	3
Chronic obstructive pulmonary disease	0
Others	29
No comorbids	146 (73.4)
Outcome	
Discharged	196 (98.5)
Deceased	1 (0.5)
Transfer out	2 (1)

*n = 199 for all variables except for BMI (*n* = 109), cigarette smoking (*n* = 124), and alcohol consumption (*n* = 62)

Cough and fever were the most prevalent symptom affecting 45 (22.6%) and 40 (20.1%) patients respectively. In addition, non-specific and pleuritic chest pain were the most common atypical symptoms affecting 21 patients (10.5%) followed by headache and anosmia. Atypical symptoms like headache, giddiness, behavioural changes were more commonly seen in the elderly population. Table 2 summarises the clinical manifestations present on admission as well as during admission.

**Table 2:** Clinical manifestation amongst patients with symptoms upon presentation and during hospital admission (*n* = 96)

Parameters / Symptoms	Symptoms on presentation	Symptoms during admission	Total	*p*-value
	*n* = 53 (%)	*n* = 43 (%)	n = 96 (%)	
Cough	32 (16.1)	13 (6.5)	45	0.019*
Productive	10 (5)	1 (0.5)	11	–
Breathlessness	11 (5.5)	5 (2.5)	16	0.059
Temperature < 37.8ºC	25 (12.6)	2 (1.0)	27	0.704
Temperature > 37.8ºC	12 (6)	1 (0.5)	13	0.551
Headache	6 (3)	1 (0.5)	7	0.791
Giddiness	2 (1)	0 (0)	2	0.881
Lethargy	5 (2.5)	0 (0)	5	0.825
Body Weakness	0 (0)	1 (0.5)	1	0.482
Anosmia	11 (5.5)	0 (0)	11	0.550
Low Glasgow Coma Scale Score	1 (0.5)	0 (0)	1	0.360
Nausea	1 (0.5)	2 (1.0)	3	0.345
Vomiting	0 (0)	1 (0.5)	1	0.482
Abdominal Pain	3 (1.5)	3 (1.5)	6	0.113
Rash	0 (0)	0 (0)	0	–
Diarrhoea	4 (2)	6 (3.0)	10	0.831
Chest Pain	9 (4.5)	12 (6)	21	0.005*
Sore Throat	17 (8.5)	6 (3.0)	23	0.385
Rhinorrhoea	20 (10.1)	3 (1.5)	23	0.012*
Myalgia / Arthralgia	5 (2.5)	1 (0.5)	6	0.336

*Chi square test was used, accepting a *p*-value of < 0.05 as significant

**Fig. 1:**
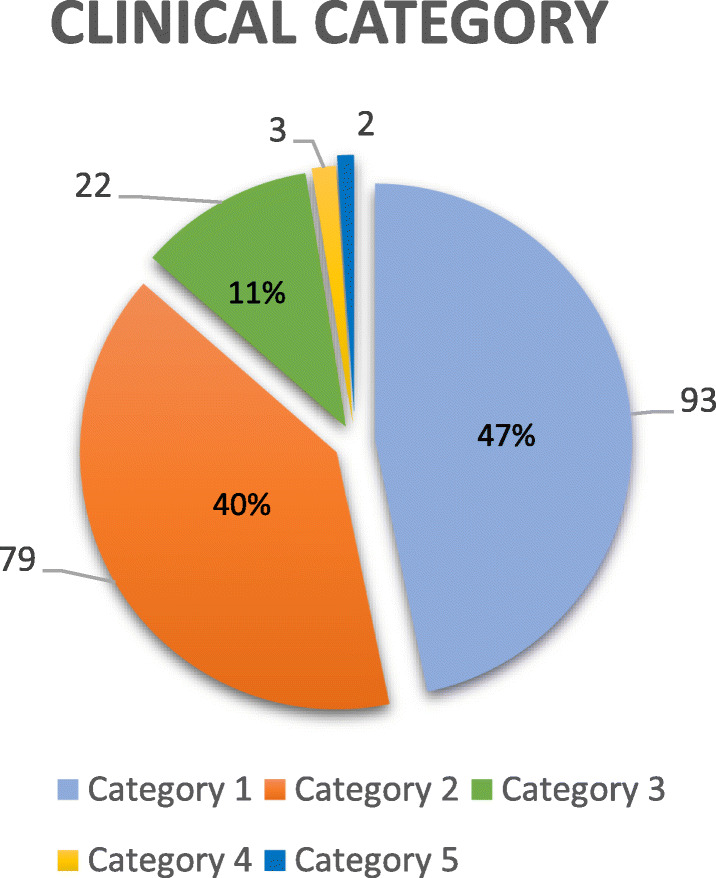
Clinical category of our total cohort of COVID-19 patients with percentages (n=199)

Nearly half of our samples were clinical category one; 93 patients (46.7%), followed by 79 patients (39.7%) with category two, 22 patients (11.1%) with category 3, 3 and 2 patients respectively with category 4 and 5. The distribution of our patient’s clinical category is illustrated in Fig. 1. In addition, the highest frequency of MEWS documented were score 1 and 2 with 157 (79.3%) and 32 (16.2%) respectively.

In terms of blood abnormalities, 50 (25.1%) patients had blood abnormalities on admission while 55 (27.6%) patients in total had abnormalities during admission. The most common abnormality noted was raised liver transaminases. Notably, one quarter of all patients had bilirubin levels at the upper limit of normal at some point during admission regardless of symptoms. All patients had chest X-rays with 30 patients demonstrating radiological abnormalities including peripheral air space opacity and/or consolidation. 44 patients had computed tomography of the thorax demonstrating peripheral consolidation in only 20 patients. 45 patients were given medications during their stay; 43 given hydroxychloroquine, 14 administered azithromycin, 4 given kaletra (lopinavir/ritonavir), 2 on interferon and one given tocilizumab. 6 patients from the asymptomatic group received hydroxychloroquine initially due to suspicious chest X-ray findings prior to reporting.

**Fig. 2:**
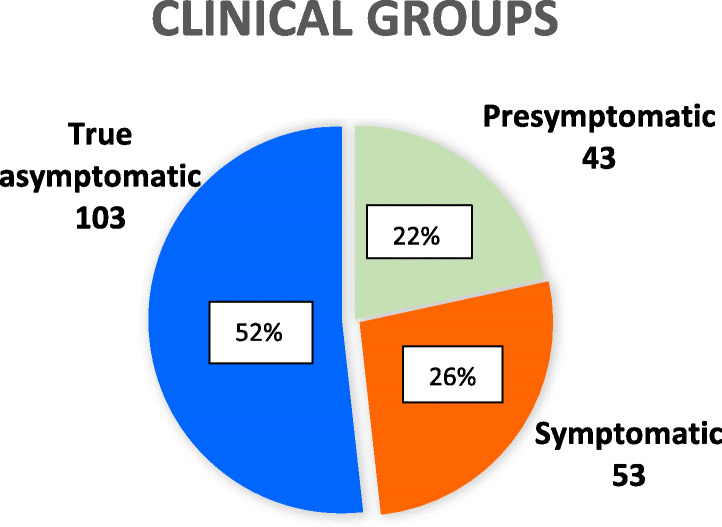
Clinical groups based on presence of symptoms amongst our cohort of COVID-19 patients with percentages (n=199)

Based on our sample, 43 (21.6%) patients were presymptomatic, 53 (26.6%) were symptomatic while 103 (51.8%) were true asymptomatics. There were 9 paucisymptomatic patients each in the symptomatic and presymptomatic groups while there were 10 (23.3%) and 5 (9.4%) patients presenting with atypical symptoms in the presymptomatic and symptomatic group respectively. Presymptomatic patients in our cohort presented with symptoms from day 1 to 14 of admission with a mean of 4 ± 2.69 (SD) day of admission and 7.37 ± 4.32 (SD) days after first positive COVID-19 RT-PCR test with a range of 1 to 24 days. Three outliers manifested symptoms beyond 14 days; 2 at day 17 and one at day 24. Figure 2 demonstrates the different clinical groups in our cohort.

Upon comparing both groups, there was a higher proportion of symptomatic patients with cough (*p* = 0.019) and rhinorrhoea (*p* = 0.012) that was found to be significant. On the other hand, chest pain was more prevalent in the presymptomatic group (*p* = 0.005). The proportion of males and females among the different clinical groups were significantly different (*p* = 0.019) with a larger percentage of females in the presymptomatic group compared to males. In general, patients from the presymptomatic group received treatment more than other groups (*p* < 0.001); majority on hydroxychloroquine. Further analysis between genders did not show any significant association with regards to blood and chest x-ray abnormalities.

Besides that, there were four patients with clinical category four and above in the symptomatic group with only one in the presymptomatic group with category four. A larger proportion of patients in the presymptomatic group had higher overall MEWS and this was statistically significant (*p* = 0.023). With regards to the time from positive to negative COVID-19 RT-PCR, the mean day to negative PCR was 16.42 ± 5.50 (SD) day of illness. There was a statistically significant difference between the presymptomatic, symptomatic and true asymptomatic groups as determined by one-way ANOVA (F(2,193) = 5.945, *p* = .003). A Tukey post hoc test revealed that the mean day to negative PCR in the symptomatic group was significantly longer in the symptomatic group (18.37 ± 7.37 days, *p* = .002) compared to the true asymptomatic group (15.26 ± 3.80 days). However, there were no statistically significant difference between the presymptomatic group compared to the symptomatic (*p* = .390) and true asymptomatic group (*p* = .219).

Table 3 summarises the comparison of the different clinical groups with multiple variables while Table 4 depicts the association between clinical characteristics and presymptomatic patients.

**Table 3:** Comparing presymptomatic, symptomatic and true asymptomatic group (*n* = 199) of patients with COVID-19

Characteristics		Presymptomatic ***n*** = 43 (%)	Symptomatic ***n*** = 53 (%)	True asymptomatic ***n*** = 103 (%)	Total n = 199
Gender	Female	18 (34.6)	14 (26.9)	20 (38.5)	52
	Male	25 (17.0)	39 (26.5)	83 (56.5)	147
Age (years)	1–18	5 (22.7)	6 (27.3)	11 (50.0)	22
	19–35	20 (18.1)	28 (25.5)	62 (56.4)	110
	36–65	15 (28.3)	14 (26.4)	24 (45.3)	53
	> 65	3 (21.4)	5 (35.7)	6 (42.9)	14
BMI**	< 18.5	1 (14.2)	3 (42.9)	3 (42.9)	7
	18.5–22.9	12 (30.0)	4 (10.0)	24 (60.0)	40
	23–26.9	5 (15.6)	14 (43.8)	13 (40.6)	32
	> 27	9 (30.0)	5 (16.7)	16 (53.3)	30
Nationality	Local	29 (20.7)	39 (27.9)	72 (51.4)	140
	Foreigner	14 (23.7)	14 (23.7)	31 (52.5)	59
Highest MEWS;	0	1 (50)	0 (0)	1 (50)	2
	1	34 (21.7)	34 (21.7)	89 (56.6)	157
	2	5 (15.6)	15 (46.9)	12 (37.5)	32
	3	3 (50)	3 (50)	0 (0)	6
	4	0 (0)	0 (0)	1 (100)	1
Category after re-evaluation	1	0 (0)	1 (1.1)	92 (98.9)	93
	2A	27 (45.8)	32 (54.2)	0 (0)	59
	2B	8 (40.0)	12 (60.0)	0 (0)	20
	3A	5 (25.0)	4 (20.0)	11 (55.0)	20
	3B	2 (100.0)	0 (0)	0 (0)	2
	4A	1 (50.0)	1 (50.0)	0 (0)	2
	4B	0 (0)	1 (100.0)	0 (0)	1
	5	0 (0)	2 (100.0)	0 (0)	2
Cigarette smoker**	Yes	2 (13.3)	5 (33.3)	8 (53.3)	15
	Reformed	1 (12.5)	4 (50.0)	3 (37.5)	8
	No	24 (23.8)	20 (19.8)	57 (56.4)	101
Blood abnormalities on admission	Yes	10 (20.0)	15 (30.0)	25 (50.0)	50
	No	33 (22.2)	38 (25.5)	78 (52.3)	149
Blood abnormalities during stay	Yes	11 (20.0)	18 (32.8)	26 (47.2)	55
	No	32 (22.2)	35 (24.3)	77 (53.5)	144
Abnormal blood indices	CBC RP LFT	2 (33.3) 4 (20.0) 11 (20.0)	1 (16.7) 6 (30.0) 20 (36.4)	3 (50.0) 10 (50.0) 24 (43.6)	6 20 55
X-ray changes	Yes	10 (33.3)	9 (30.0)	11 (36.7)	30
	No	33 (19.5)	44 (26.0)	92 (54.4)	169
CT thorax performed	Yes	8 (18.2)	11 (25.0)	25 (56.8)	44
	No	35 (22.6)	42 (27.1)	78 (50.3)	155
Treatment	Yes	20 (44.4)	19 (42.2)	6 (13.3)	45
Days to negative PCR	Mean ± SD (Days)	16.9 ± 5.78	18.37 ± 7.37	15.26 ± 3.80	16.42 ± 5.50 *p* = 0.003***

*Percentages calculated for each row equivalent to 100%

**n = 199 for all variables except for BMI (n = 109), cigarette smoking (n = 124) and days to negative PCR (*n* = 196)

***one-way ANOVA whereby the mean difference is significant at the 0.05 level

**Table 4:** Association between clinical characteristics and presymptomatic COVID-19 patients in our cohort

Characteristics		n (%)	Presymptomatic		*P* value
			Yes (%)	No (%)	
Gender	Female	52 (26.1)	18 (34.6)	34 (65.4)	0.019*
	Male	147 (73.9)	25 (17.0)	122 (83.0)	
Nationality	Local	140 (70.4)	29 (20.7)	111 (79.3%)	0.707
	Foreigner	59 (29.6)	14 (23.7)	45 (76.3)	
Highest MEWS;	0	2 (1.0)	1 (50.0)	1 (50.0)	0.023*
	1	157 (79.3)	34 (21.7)	123 (78.3)	
	2	32 (16.2)	5 (15.6)	27 (84.4)	
	3	6 (3.0)	3 (50.0)	3 (50.0)	
	4	1 (0.5)	0 (0)	1 (100.0)	
Category after re-evaluation	1	93 (46.7)	0 (0)	93 (100.0)	–
	2A	59 (29.6)	27 (45.8)	32 (54.2)	
	2B	20 (10.1)	8 (40.0)	12 (60.0)	
	3A	20 (10.1)	5 (25.0)	15 (75.0)	
	3B	2 (1.0)	2 (100.0)	0 (0)	
	4A	2 (1.0)	1 (50.0)	1 (50.0)	
	4B	1 (0.5)	0 (0)	1 (100.0)	
	5	2 (1.0)	0 (0)	2 (100.0)	
Cigarette smoking	Yes	15 (12.1)	2 (13.3)	13 (86.7)	0.359
	Reformed	8 (6.4)	1 (12.5)	7 (87.5)	
	No	101 (81.5)	24 (23.8)	77 (76.2)	
Blood abnormalities on admission	Yes	50 (25.1)	10 (20.0)	40 (80.0)	0.817
	No	149 (74.9)	33 (22.1)	116 (77.9)	
Blood abnormalities during stay	Yes	55 (27.6)	11 (20.0)	44 (80.0)	0.485
	No	144 (72.4)	22 (22.2)	112 (71.8)	
Abnormal blood indices	CBC RP LFT	6 (3.0)** 20 (10.1)** 55 (27.6)**	2 (33.3) 4 (20.0) 11 (20.0)	4 (66.7) 16 (80.0) 44 (80.0)	0.442 0.487 0.119
X-ray changes	Yes	30 (15.1)	10 (33.3)	20 (66.7)	0.126
	No	169 (84.9)	33 (19.5)	136 (80.5)	
CT thorax performed	Yes	44 (22.1)	8 (18.6)	36 (23.1)	0.748
	No	155 (77.9)	35 (81.4)	120 (76.9)	
Treatment	Yes	45 (22.6)**	20 (44.4)	25 (55.6)	< 0.001*
Negative PCR based on day of illness	Mean	16.42 Days	17 Days	16 Days	0.293

*Fisher exact test with level of significance at < 0.05

**Percentages depicting total number of abnormal readings/treated patients only (n = 199)

Otherwise, no significant difference was observed related to categories, smoking history, blood abnormalities, and radiological findings among patients with different clinical groups. Table 5 compare the variables between genders.

**Table 5:** Statistical comparison between genders and clinical variables in our cohort of COVID-19 patients

Variables	Male (%) *n* = 147	Female (%) *n* = 52	*P*-value
Blood abnormalities on admission	40 (27.2)	10 (19.2)	0.254
Blood abnormalities during stay	41 (27.9)	14 (26.9)	0.893
Chest x-ray abnormality	20 (13.6)	10 (19.2)	0.330
Treatment given	27 (18.4)	18 (34.6)	0.016^*^

*The Chi-square statistic is significant at the .05 level

We had a large number of foreign nationals admitted; 59 (29.6%) patients in total; comprising of Indonesians, Bangladeshis, Pakistanis, Indian and Myanmar nationals. From this diverse demography, 14 patients each were presymptomatic and symptomatic while 31 were true asymptomatic. Besides that, 4 of these patients were clinically category 3 but none were severely ill.

## Discussion

Our study of 199 patients revealed a relatively large proportion of presymptomatic patients; amounting to 43 (21.6%); interestingly with a female preponderance. This may be related to studies suggesting higher circulating angiotensin-converting enzyme 2 in male patients leading to infection and possibly higher viral loads causing clinical manifestation, but more research is required before jumping into conclusions seeing that a large proportion of male patients were asymptomatic as well [13]. Conversely, females were initiated on treatment more frequently. This could be explained by the higher proportion of presymptomatic presentation, giving the impression of disease progression during admission. Published data have suggested a higher infection rate and mortality in male patients and in our cohort, we did observe higher admissions and one death among male patients [14]. However, there are no significant statistical difference in terms of investigation results or ill patients upon comparing both genders.

Apart from that, the typical symptoms of cough and rhinorrhoea were evidently the most frequent symptoms observed in our sample as well. Respiratory symptoms like cough, shortness of breath, sore throat and rhinorrhoea were more common in the symptomatic group likely due to COVID-19 being educated to the public as a respiratory disease as well as being severe enough to prompt them to seek medical advice. Atypical chest pains presented more frequently in the presymptomatic group and whether this may be due to inflammatory responses or viral shedding needs to be looked into [15]. MEWS was generally higher in the symptomatic group as well due to fever and increased respiratory rate as a physiological compensatory mechanism to metabolic acidosis secondary to pyrexia.

We observe a lengthier time to negative conversion of initial PCR positive result among symptomatic patients compared to those who were asymptomatic. Lee et al. had demonstrated through analysis of the cycle threshold value dynamics of the RdRp gene that the viral load decreased at a slower rate among presymptomatic and symptomatic patients than asymptomatic patients [16]. However, the opposite was seen when the envelope gene was analysed. Regardless, the persistence of viral molecular shedding did not equate to a persistent infectious state.

As a result, this further highlights the difficulty in identifying these ‘silent spreaders’ who will escape detection despite being at the peak of viral shedding. If the numbers are to be taken literally, over 20% of infected patients may be actively shedding the virus undetected despite point of entry screenings. However, as keeping the public at home at all times is impractical, additional steps must be taken to contain the disease effectively via primary prevention.

A review on 172 studies revealed that maintaining a physical distance of one meter reduces transmission rates significantly; more so if a distance of two meters and above is kept [17]. Besides that, the use of face masks resulted in a large reduction in risk of infection; significantly greater with the use of 3-ply surgical masks and N95 respirators. However, this would not be practical with the global shortage of N95 masks for the time being and should be reserved for high-risk professions and aerosol generating procedures as recommended by the Centers for Disease Control and Prevention (CDC) [18].

Droplet precaution also includes frequent hand washing with correct techniques and sufficient duration with soap or alcohol-based sanitizers. Its transmissibility via fomites is well documented and eye or face shields would further curb the spread in public and especially in an outpatient clinic setting [19–21]. Aside from strictly limiting visitors to healthcare facilities, there should be a movement towards the direction of telemedicine to reduce outpatient numbers without compromising care.

Two other important strategies are contact tracing and mass screening. However, contact tracing’s effectiveness may be hampered by misinformation and its slow process, in which several ‘generations’ of disease transmission may have already occurred. In this digital age, applications like ‘TraceTogether’ and ‘CovidSafe’ in Singapore and Australia have tremendously increased the speed of tracing with the focus on significant person to person contact recorded via Bluetooth; storing information on mobile phones [22]. In addition, applications like ‘NZ Covid Tracer’ and ‘MySejahtera’ from New Zealand and Malaysia respectively allows users to check in locations via QR codes, negating the need for manual documentation in which stationaries may be shared and allows contact tracing to be rapid in instances where a positive patient may have visited previously [23].

By now, South Korea’s success story with mass screening as its backbone is well documented; testing close to a quarter million people in just one and a half months allowing rapid isolation of those affected [24]. High risk populations should be frequently screened including nursing centres and establishments in which close contacts are unavoidable like hostels, prisons and detention centres. Malaysia; taking a leaf out of Singapore’s book on handling migrant workers; had detected a large proportion of patients by screening documented and undocumented foreign workers as evident in our cohort, making up one third of total patients [25]. In addition, South Korea have implemented low-contact testing centres allowing nasopharyngeal swabs to be performed from booths and via drive-throughs which is also practiced locally here in government and private healthcare facilities [26].

In efforts to further reduce contact, several large corporations have implemented guidelines allowing employees to work from home whenever possible [27]. This reduces the risk of transmission but may only be practical for white collar workers. Therefore, to cater to other working classes, countries like Germany have provided financial aid by directly paying a large percentage of the wages of their working class, thereby greatly reducing unemployment and retrenchment with an overall cost benefit of not needing to retrain new workers [28].

There were several limitations to our study. Firstly, we were not able to assess our cohort of presymptomatic patients’ quantitative RT-PCR levels prior and during symptom onset and that would be able to shed light on the temporal dynamics in viral shedding as illustrated by He and Lau et al. [5] The result obtained may support the notion that the peak period of viral shedding occurs 1–2 days prior to symptom onset, increasing infectivity and highlighting the importance of this period. Secondly, as the history was obtained through medical records and clarified with patients, there may be errors in ascertaining the exact date of symptoms and clinical manifestation which might inadvertently lead to patients being classified wrongly. Thirdly, in accordance to our local guidelines, we had repeated swabs at day 13 of illness and if positive, 48 to 72 h subsequently based on updates in the guidelines. Therefore, we could not accurately portray the length of positive PCR results as some patients might have demonstrated a negative result prior to day 13 of illness.

## Conclusion

The Covid-19 pandemic, being a year old still carries a lot of unanswered questions. A large proportion of patients are presymptomatic and capable of spreading the infection without any obvious early indication of illness. While researchers are still coming up with definitive treatments, vaccines, and an ideal rapid test that would aid us in treating and identifying these silent spreaders, we need to buy time by reducing the risk of transmission. Still, lengthy lockdowns have a negative socio-economic impact but nations can replicate countries like Germany and South Korea to name a few who have been able to remain open with little restrictions, minimising the economic impact of the pandemic without compromising healthcare. In the meantime, buzzwords like ‘social distancing’, ‘social responsibility’ and ‘new normal’ must constantly play in the publics’ minds to remind us to adopt new practices to keep our community safe and break the chain of transmission.

## Data Availability

The datasets used and/or analysed during the current study are available from the corresponding author on reasonable request.

## Abbreviations

COVID-19: Coronavirus disease 2019
SARS-CoV-2: Severe acute respiratory syndrome coronavirus 2
RT-PCR: Reverse transcription polymerase chain reaction
RdRp: RNA-dependent RNA polymerase
MEWS: Modified early warning score
ANOVA: Analysis of variance
BMI: Body mass index
SD: Standard deviation
